# Reliability of information recorded on the National Immunization Program Information System

**DOI:** 10.1590/S2237-96222024v33e20231309.especial2.en

**Published:** 2024-10-21

**Authors:** José Cássio de Moraes, Ana Paula França, Ione Aquemi Guibu, Rita Barradas Barata, ICV 2020 Group, Adriana Ilha da Silva, Alberto Novaes Ramos, Ana Paula França, Andrea de Nazaré Marvão Oliveira, Antonio Fernando Boing, Carla Magda Allan Santos Domingues, Consuelo Silva de Oliveira, Ethel Leonor Noia Maciel, Ione Aquemi Guibu, Isabelle Ribeiro Barbosa Mirabal, Jaqueline Caracas Barbosa, Jaqueline Costa Lima, José Cássio de Moraes, Karin Regina Luhm, Karlla Antonieta Amorim Caetano, Luisa Helena de Oliveira Lima, Maria Bernadete de Cerqueira Antunes, Maria da Gloria Teixeira, Maria Denise de Castro Teixeira, Maria Fernanda de Sousa Oliveira Borges, Rejane Christine de Sousa Queiroz, Ricardo Queiroz Gurgel, Rita Barradas Barata, Roberta Nogueira Calandrini de Azevedo, Sandra Maria do Valle Leone de Oliveira, Sheila Araújo Teles, Silvana Granado Nogueira da Gama, Sotero Serrate Mengue, Taynãna César Simões, Valdir Nascimento, Wildo Navegantes de Araújo

**Affiliations:** 1 Faculdade de Ciências Médicas da Santa Casa de São Paulo, Departamento de Saúde Coletiva, São Paulo, SP, Brasil; Universidade Federal do Espírito Santo, Vitória, ES, Brazil; Universidade Federal do Ceará, Departamento de Saúde Comunitária, Fortaleza, CE, Brazil; Faculdade Ciências Médicas Santa Casa de São Paulo, São Paulo, SP, Brazil; Secretaria de Estado da Saúde do Amapá, Macapá, AP, Brazil; Universidade Federal de Santa Catarina, SC, Brazil; Organização Pan-Americana da Saúde, Brasília, DF, Brazil; Instituto Evandro Chagas, Belém, PA, Brazil; Faculdade de Ciências Médicas Santa Casa de São Paulo, Departamento de Saúde Coletiva, São Paulo, SP, Brazil; Universidade Federal do Rio Grande do Norte, Natal, RN, Brazil; Universidade Federal do Ceará, Departamento de Saúde Comunitária, Fortaleza, CE, Brazil; Universidade Federal de Mato Grosso, Cuiabá, MT, Brazil; Universidade Federal do Paraná, Curitiba, PR, Brazil; Universidade Federal de Goiás, Goiânia, GO, Brazil; Universidade Federal do Piauí, Teresina, PI, Brazil; Universidade de Pernambuco, Faculdade de Ciências Médicas, Pernambuco, PE, Brazil; Instituto de Saúde Coletiva, Universidade Federal da Bahia, Salvador, BA, Brazil; Secretaria de Estado da Saúde de Alagoas, Maceió, AL, Brazil; Universidade Federal do Acre, Rio Branco, AC, Brazil; Universidade Federal do Maranhão, Departamento de Saúde Pública, São Luís, MA, Brazil; Universidade Federal de Sergipe, Aracaju, SE, Brazil; Secretaria Municipal de Saúde, Boa Vista, RR, Brazil; Fundação Oswaldo Cruz, Mato Grosso do Sul, Campo Grande, MS, Brazil; Fundação Oswaldo Cruz, Escola Nacional de Saúde Pública Sergio Arouca, Rio de Janeiro, RJ, Brazil; Universidade Federal do Rio Grande do Sul, Porto Alegre, RS, Brazil; Fundação Oswaldo Cruz, Instituto de Pesquisa René Rachou, Belo Horizonte, MG, Brazil; Secretaria de Desenvolvimento Ambiental de Rondônia, Porto Velho, RO, Brazil; Universidade de Brasília, Brasília, DF, Brazil

**Keywords:** Sistema de Información en Salud, Confiabilidad, Vacunas, Health Information Systems, Reliability, Vaccines

## Abstract

**Objective:**

To analyze the reliability of records held on the National Immunization Program Information System (SI-PNI) in a subsample of children included in the national vaccination coverage survey in Brazilian state capitals and Federal District in 2020.

**Methods:**

This was a study of agreement between data recorded on vaccination cards (doses and dates) and on the SI-PNI for 4050 children with full coverage at 24 months.

**Results:**

Data on 3587 children were held on the SI-PNI, with losses of 11% (95%CI: 10;12). Total agreement between doses and dates in the two sources was 86% (95%CI: 86;87), however taking each dose and vaccine individually, variation was greater, with 32% of data in only one source.

**Conclusion:**

Part of the information was not recorded, but the discrepancy can be considered small. Nonetheless, underrecording of doses and children can compromise vaccination coverage estimates, altering the numerator and denominator data.

## Study contributions

**Main results:**

Subsample of 4,050 children, among those completing the full schedule at 24 months studied in the national survey, 11% had not been recorded on the SI-PNI, 32% had unrecorded doses (doses or dates) and there was 8% disagreement between vaccination cards and SI-PNI records.

**Implications for services:**

Recognizing the difficulties faced by the SI-PNI and the discrepancies between sources is essential for adopting initiatives to improve data quality, so as to avoid inaccurate estimates of childhood vaccination coverage.

**Perspectives:**

This study is expected to contribute to improving the quality of records and the usability of data for monitoring vaccination coverage of the immunization program from the local to the national level.

## RESUMEN

**Objetivo:**

Evaluar la confiabilidad de los registros del Sistema de Información del Programa Nacional de Vacunación (SI-PNI), en una submuestra de niños incluidos en la encuesta nacional de cobertura vacunal en capitales de Brasil y Distrito Federal, en 2020 .

**Método:**

Estudio de concordancia entre datos en carné vacunal (dosis y fechas) y en el SI-PNI para 4.050 niños con pauta completa a los 24 meses.

**Resultados:**

Se ubicaron 3.587 niños en el SI-PNI con un 11% (IC95% 10,0;12,0) de pérdidas. La concordancia total entre dosis y fechas en las dos fuentes fue del 86% (IC95% 86,0;87,0), pero por dosis y vacuna la variación fue mayor, con 32% de datos en una única fuente.

**Conclusión:**

Parte de la información no se ha registrado, pero para los datos existentes en ambas fuentes, la discrepancia puede considerarse pequeña. El subregistro de dosis y de niños puede comprometer las estimaciones de cobertura vacunal.

## INTRODUCTION

The National Immunization Program (*Programa Nacional de Imunizações* - PNI) is the key element in the control of vaccine-preventable diseases, namely the set of illnesses for which vaccines have been developed as primary prevention instruments, that is, capable of preventing infection or the development of diseases in those immunized against them. The technological arrangement adopted to control this group of communicable diseases combines a series of elements, such as routine vaccination, national vaccination days, occasional campaigns and epidemiological surveillance.^1^

Routine vaccination consists of establishing a national vaccination schedule containing all vaccines that should be offered to different age groups of the population, indicating the number of doses for each vaccine, as well as the correct ages for administration (epidemiological adequacy) and the correct intervals between doses (immunological adequacy) with the intention of obtaining sufficient population coverage so that mass or herd immunity can also function effectively as a protection mechanism for those who have not been vaccinated, thus keeping the circulation of etiological agents under control.^1^

National vaccination days, organized in the form of a large-scale action carried out twice a year to expand and facilitate access to those families who, for different reasons, have difficulties in regularly using health services, represented for many years a strategy of positive discrimination, aiming to provide equitable opportunities for vaccinating all population groups in the country, in addition to routine activities.^2^

Occasional vaccination campaigns, or so-called transmission-blocking vaccination, occur when outbreaks or cases of vaccine-preventable diseases are identified, in delimited space-time clusters, serving as a supplementary mechanism in the protection of particular population groups or segments. Generally, these actions are linked to the epidemiological surveillance system which, upon detecting suspected or confirmed cases of these diseases, triggers various contention actions to interrupt their transmission.

Monitoring the vaccination status of children under 24 months of age is particularly important due to the relevance that vaccine-preventable diseases have on morbidity and mortality in childhood, both in those under 1 year old (infant mortality) and in those under 5 years old. Therefore, information on vaccination coverage in childhood is essential for analyzing the health situation and steering child health policies.

The National Immunization Program Information System (SI-PNI) is made up of a set of systems in order to meet all aspects involved in carrying out the national immunization program: evaluation of the immunization program, stock and distribution of immunobiologicals, post-vaccination adverse events, supervision instrument evaluation program, vaccination room supervision instrument evaluation program, control of immunobiologicals used and the Special Immunobiological Reference Center information system.^3^ This study only involves the immunization program evaluation system.

Prior to the creation of the SI-PNI, vaccination coverage was expressed by the number of doses administered for a given vaccine over the recorded or estimated number of children under 1 year old or under 24 months old within the program’s coverage area. This way of estimating coverage usually generated overestimated values for each vaccine, and did not allow coverage to be assessed based on the full schedule, since the information had varying degrees of imprecision, in addition to not corresponding to the experience of each child in the age group of interest.^1^

The SI-PNI was designed to allow longitudinal monitoring of each child included in the program, in addition to allowing vaccines administered in different health services to be recorded on the same online vaccination record. Each vaccine dose should be recorded on the child’s vaccination card and also on the information system, guaranteeing a reliable and double record of the doses administered, this being essential for increasing the security of the records of each person’s vaccination history. In addition to providing a tool for analyzing vaccination coverage at different levels of analysis (health service, district, municipality, state and country), the system enables recovery of each child’s vaccination history even if the physical document, the vaccination card, is lost or damaged.^4^

The introduction of the new information system, which began in 2010, progressively achieved coverage over the following five years, coinciding with a period of reduction in childhood vaccination coverage in Brazil, giving rise to several doubts about the reasons for such a reduction, including the possible role of the new information system. Taking into account the geographic size of Brazil and the known difficulties relating to internet networks in different regions of the country and the slowness that a national system can present in recording information, one of the issues raised was the possible delay in recording and sending information from the local level, resulting in an apparent reduction in coverage.

The occurrence of measles epidemics, however, seemed to indicate a real reduction in coverage, regardless of problems with the recording system. Therefore, it was necessary to assess the extent to which recording on the SI-PNI was being properly carried out.

The objective of this study was to analyze the reliability of the records kept on the immunization program evaluation system, part of the SI-PNI, for a subsample of children included in the national vaccination coverage survey carried out in Brazilian state capitals and Federal District, in 2020, with full coverage at 24 months of age. The research questions were: 

*How many of these children were recorded on the SI-PNI? For those recorded, how much agreement was there between vaccination card data and SI-PNI data?*

## METHODS

Carrying out the national vaccination coverage survey in the Brazilian state capitals and Federal District offered the opportunity to analyze a subsample of children born in 2017 and 2018, living in the urban areas of these cities, comparing the data available on their vaccination cards with SI-NIP data. The survey methodology is described in detail in another publication.^5^

The target population of this reliability study were children participating in the national survey who had full coverage for vaccines scheduled up to 24 months old, according to documentation, doses and administration dates on their vaccination cards. Therefore, vaccination card records were considered to be the gold standard. The vaccination cards were photographed during the home interviews and the data were later added to each child’s questionnaire, under the supervision of a nursing professional with National Immunization Program experience.

Of the 31,001 children included in the national survey, 18,808 (58%) had full vaccination coverage. Of this total, 150 children residing in each of the state capitals and the Federal District were selected randomly, so that a subsample of 4,050 children was obtained. This strategy allowed comparisons to be made as to the quality of data entry on the SI-PNI between the state capitals.

The sample size was defined arbitrarily, considering the work involved in locating the children on the SI-PNI and transcribing the data for subsequent analysis.

For each of the 4,050 children in the subsample, the corresponding records on the SI-PNI were searched for based on the mother’s name and the child’s date of birth. Both the doses and dates recorded on the vaccination cards and the corresponding records on the SI-PNI were input to the study database. 

The data were compared regarding the dose and date records contained in the two sources of information. For each dose, the results were classified into three groups: agreement between the dates recorded on both instruments, disagreement between the dates recorded on one of the instruments and records existing only on the vaccination card and not appearing on the information system. Percentages and 95% confidence intervals (95%CI) were calculated.

The percentages of agreement between doses and dates in the two sources of information were compared, separately, for children who used public vaccination services and for those who received at least one dose in private services. Association was tested using the chi-square method.

The distribution of percentage agreement between the data in the two sources, for each of the vaccine doses applied to each child, in each city, was analyzed using boxplot graphs. Boxplot graphs present a summary of distribution representing the median value and the interquartile range in the boxes (box) and outliers, plus or minus, in graphic signs presented outside the boxes. Thus, it was possible to compare the values found for each state capital regarding agreement between the two sources of information.

For each of the 150 children in each city, the combined agreement of doses that make up the recommended schedule for the first 24 months of life was analyzed. This involved three doses of DTcP-Hib-Hepb vaccine (considering all its available presentations), three doses of inactivated poliomyelitis vaccine, two doses and one booster of antipneumococcal vaccine, two doses and one booster of antimeningococcal C vaccine, two doses of rotavirus vaccine, the first dose of measles, mumps and rubella (MMR) vaccine, hepatitis A vaccine and varicella vaccine. We did not include Bacillus Calmette-Guérin (BCG) and hepatitis B vaccinations, at birth, because the records were missing on the SI-PNI for the majority of the children, nor yellow fever vaccination, because it had not been implemented in all the cities in 2017 and 2018. The second MMR dose was also excluded because of the large amount of missing information on the SI-PNI.

We used Stata version 17.0 for all the analyses and for preparing the graphs.

The study was approved by the Human Research Ethics Committee of the Instituto de Saúde Coletiva of the Universidade Federal da Bahia, as per Opinion No. 3.366.818, on June 4^th^ 2019, Certificate of Submission for Ethical Appraisal (*Certificado de Apresentação de Apreciação Ética* (CAAE) No. 4306919.5.0000.5030; and by the Human Research Ethics Committee of the Santa Casa de São Paulo, as per Opinion No. 4.380.019, on November 4^th^ 2020, CAAE No. 39412020.0.0000.5479. 

## RESULTS

Of the 4,050 children selected, the corresponding records were found for 3,587 (89%) of them on the SI-PNI. As such, 11% (955CI 10,0;12,0) of the children did not have their data recorded on the system. These losses occurred in all the cities and were above 20% in São Luís, Rio de Janeiro and Campo Grande (Table 4).

Table 1 shows, for each dose of vaccine, the proportion of doses with concordant, discordant or missing records on the SI-PNI for the total of 3,587 children with information in both data sources. In general, around 32% of vaccine dose administration information was missing on the SI-PNI, 58% to 60% of the information was concordant in both sources and 8% was discordant. Table 1 also highlights the much higher percentages of missing information system records for doses of intradermal BCG, hepatitis B and MMR (second dose). 

**Table 1:** Agreement (%) between vaccination card information and SI-PNI records, according to vaccine dose as per vaccination schedule by 24 months old, National Vaccination Coverage Survey, Brazil, 2020

Vaccine	Concordant N (%)	Discordant N (%)	Missing N (%)
Intradermal BCG	1,239 (34.5)	123 (3.4)	2,223 (62.1)
Hepatitis B	999 (27.9)	135 (3.8)	2,449 (68.3)
DTcP-Hib-Hepb 1^st^ dose	2,211 (61.6)	218 (6.1)	1,155 (32.3)
DTcP-Hib-Hepb 2^nd^ dose	2,177 (60.7)	254 (7.1)	1,147 (32.2)
DTcP-Hib-Hepb 3^rd^ dose	2,176 (60.7)	277 (7.7)	1,077 (31.6)
Inactivated poliomyelitis 1^st^ dose	2,276 (63.4)	207 (5.8)	1,104 (30.8)
Inactivated poliomyelitis 2^nd^ dose	2,220 (61.9)	249 (6.9)	1,115 (31.2)
Inactivated poliomyelitis 3^rd^ dose	2,177 (60.7)	262 (7.3)	1,099 (32.0)
Pneumococcal 1^st^ dose	2,166 (60.4)	285 (7.9)	1,136 (31.7)
Pneumococcal 2^nd^ dose	2,138 (59.6)	283 (7.9)	1,166 (32.5)
Pneumococcal booster	2,230 (62.1)	296 (8.3)	1,056 (29.6)
Rotavirus 1^st^ dose	2,232 (62.3)	252 (7.0)	1,075 (30.7)
Rotavirus 2^nd^ dose	2,148 (59.9)	242 (6.7)	1,149 (33.4)
Meningococcal 1^st^ dose	2,180 (60.8)	327 (9.1)	1,080 (30.1)
Meningococcal 2^nd^ dose	2,107 (58.8)	320 (8.9)	1,157 (32.3)
Meningococcal booster	2,208 (61.5)	282 (7.9)	995 (30.6)
Hepatitis A	2,401 (66.9)	250 (7.0)	934 (26.1)
MMR 1^st^ dose	2,344 (65.4)	536 (14.9)	706 (19.7)
MMR 2^nd^ dose	951 (26.5)	134 (3.7)	619 (69.8)
Varicella	2,344 (65.4)	262 (7.3)	971 (27.3)
Oral poliomyelitis 1^st^ booster	2,255 (62.8)	401 (11.2)	878 (26.0)
DTP 1^st^ booster	2,088 (58.2)	297 (8.3)	953 (33.5)

Table 2 shows the comparison of concordant records for children who received all doses in public services and for those who received at least one dose in private services. In general, there was greater agreement when vaccines were administered in public services, with the exception of BCG, the first dose of rotavirus vaccine, and the second dose of MMR vaccine. In the case of hepatitis B vaccine, although estimated agreement was greater among children who used private services, the difference was not statistically significant.

**Table 2:** Agreement (%) between vaccination card information and SI-PNI records, according to vaccine dose as per vaccination schedule by 24 months old, for children vaccinated exclusively, or not, in public services, National Vaccination Coverage Survey, Brazil, 2020

Vaccines	Administered exclusively in public services		p-value
	**No**	**Yes**	
Intradermal BCG	279 (39.9)	938 (33.2)	0.026
Hepatitis B	219 (31.3)	766 (27.1)	0.202
DTcP-Hib-Hepb 1^st^ dose	392 (56.0)	1,786 (63.2)	0.001
DTcP-Hib-Hepb 2^nd^ dose	376 (53.7)	1,771 (62.7)	< 0.001
DTcP-Hib-Hepb 3^rd^ dose	351 (50.1)	1,790 (63.4)	< 0.001
Inactivated poliomyelitis 1^st^ dose	419 (59.9)	1,822 (64.5)	0.005
Inactivated poliomyelitis 2^nd^ dose	389 (55.6)	1,797 (63.6)	< 0.001
Inactivated poliomyelitis 3^rd^ dose	362 (51.7)	1,784 (63.2)	< 0.001
Pneumococcal 1^st^ dose	394 (56.3)	1,741 (61.7)	0.030
Pneumococcal 2^nd^ dose	393 (56.1)	1,711 (60.6)	0.011
Pneumococcal booster	365 (37.1)	1,828 (64.7)	< 0.001
Rotavirus 1^st^ dose	418 (58.4)	1,782 (63.1)	0.007
Rotavirus 2^nd^ dose	409 (58.4)	1,704 (60.3)	0.005
Meningococcal 1^st^ dose	394 (56.3)	1,751 (62.0)	0.042
Meningococcal 2^nd^ dose	370 (52.9)	1,706 (60.4)	< 0.001
Meningococcal booster	323 (46.1)	1,846 (65.4)	< 0.001
Hepatitis A	437 (62.4)	1,925 (68.2)	0.045
MMR 1^st^ dose	428 (61.1)	1,882 (66.6)	0.017
MMR 2^nd^ dose	207 (29.6)	727 (25.7)	< 0.001
Varicella	421 (60.1)	1,887 (66.8)	0.019
Oral poliomyelitis 1^st^ booster	398 (56.9)	1,819 (64.4)	< 0.001
DTP 1^st^ booster	331 (47.3)	1,726 (61.1)	< 0.001

**Figure 1:**
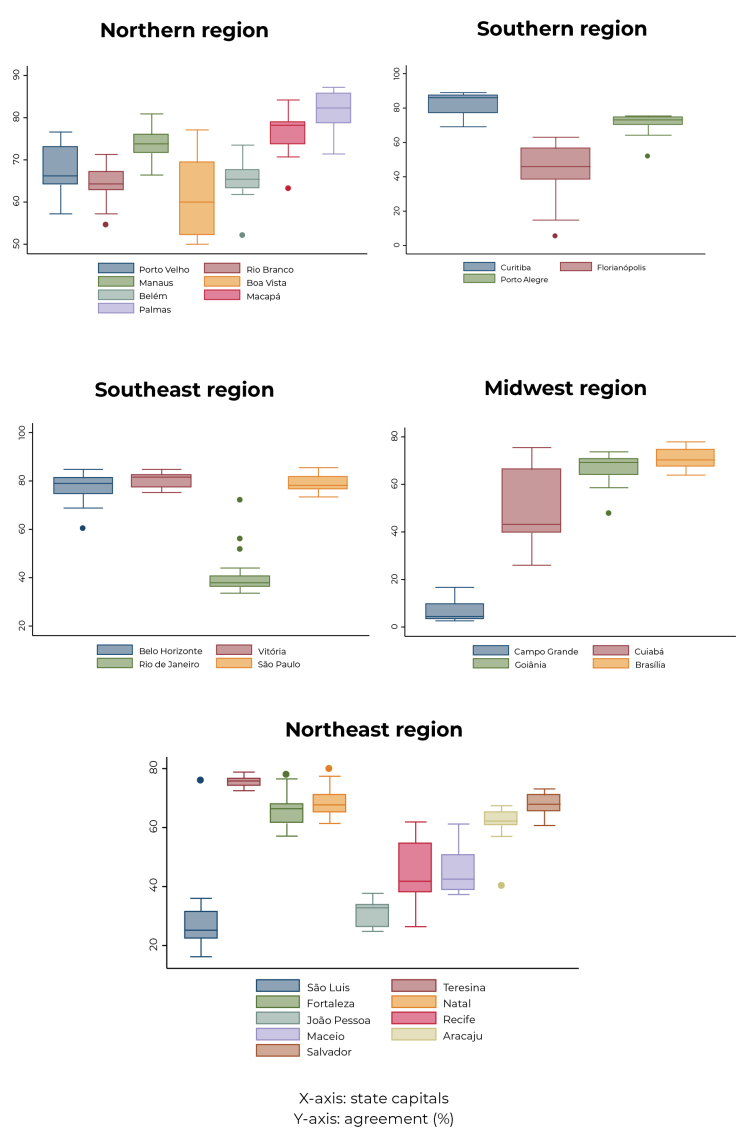
Distribution of agreement (%) for each vaccine and dose, by state capitals and the Federal District, in each Brazilian region, National Vaccination Coverage Survey, Brazil, 2020

Figure 1 shows the distribution of percentage agreement for the doses analyzed in each of the state capitals and the Federal District. The municipalities were grouped by region of the country to make viewing easier. A great variation can be seen between the municipalities in the Northeast region, with the highest agreement generally found for Teresina, Fortaleza, Natal, Aracaju and Salvador, and the lowest for São Luís and João Pessoa.

In the Northern region, Palmas and Macapá show greater agreement, while the lowest values were found for Boa Vista, with great variability in that city. In the Southeast region, the situation was more homogeneous, with the exception of Rio de Janeiro, which had lower agreement in general. In the Southern region, Florianópolis showed the lowest agreement and the highest dispersion. In the Midwest region, the poorest performance was found for Campo Grande, with Cuiabá in an intermediate position and Goiânia and Brasília with greater agreement.

Table 3 shows the extremely low proportions of agreement for the set of vaccines selected in each of the state capitals and the Federal District, when the data for each child are considered longitudinally. These data suggest that coverage calculations using the full scheme would give different results depending on the source of information used.

**Table 3:** Records missing (%) from the SI-PNI and agreement (%) between vaccine card information and SI-PNI records for the set of vaccines ^a^ comprising the vaccination schedule by 24 months old, by state capitals and the Federal District, National Vaccination Coverage Survey, Brazil, 2020

City	Missing records (95%CI)	Agreement (95%CI)
**Northern Region**		
Macapá	11.3 (7.0;17.2)	9.8 (5.0;14.5)
Belém	9.3 (5.4;14.8)	12.5 (7.2;17.8)
Rio Branco	4.7 (2.0;9.9)	13.3 (7.8;18.7)
Boa Vista	6.7 (3.4;11.6)	14.3 (8.7;19.9)
Manaus	6.0 (3.0;10.7)	16.3 (10.4;22.2)
Palmas	6.0 (3.0;10.7)	17.0 (11.0;23.0)
Porto Velho	3.3 (1.2;7.2)	17.9 (11.8;24.1)
**Northeast Region**		
Teresina	12.0 (7.5;18.0)	9.8 (5.1;14.6)
Aracaju	10.0 (6.4;16.6)	10.4 (5.5;15.2)
João Pessoa	16.7 (11.3;23.3)	11.2 (6.1;16.2)
Fortaleza	6.7 (3.4;11.7)	12.8 (7.5;18.2)
Recife	10.7 (6.4;16.5)	13.4 (8.0;18.9)
Maceió	10.7 (6.4;16.5)	14.2 (8.6;19.8)
Salvador	10.7 (6.4;16.5)	15.7 (9.8;21.5)
Natal	17.3 (11.9;24.0)	16.1 (10.2;22.0)
São Luís	26.0 (19.5;33.5)	19.8 (13.4;26.2)
**Southeast Region**		
Belo Horizonte	8.0 (4.4;13.2)	10.1 (5.3;15.0)
Rio de Janeiro	22.7 (16.5;30.0)	10.3 (5.5;15.2)
São Paulo	17.3 (11.9;24.1)	16.1 (10.2;22.0)
Vitória	16.7 (11.3;23.4)	19.2 (12.9;25.5)
**Southern Region**		
Porto Alegre	10.7 (6.4;16.7)	11.3 (6.2;16.3)
Curitiba	9.3 (5.4;14.9)	16.2 (10.3;22.1)
Florianópolis	10.0 (5.9;15.7)	16.3 (10.4;22.2)
**Midwest Region**		
Cuiabá	7.3 (3.9;12.4)	7.2 (3.1;11.4)
Goiânia	11.3 (7.0;17.3)	14.3 (8.7;19.9)
Campo Grande	24.0 (17.7;31.3)	19.3 (13.0;25.6)
**Federal District**	3.3 (1.2;7.3)	15.2 (9.4;20.9)

a) Vaccines: DTcP-Hib-Hepb, inactivated polio, pneumococcal, rotavirus, meningococcal C, MMR, hepatitis A and varicella.

## DISCUSSION

In Public Health, data are used to monitor trends and behaviors in the population distributions of events of interest, such as cases, deaths, exposures, sociodemographic characteristics, among others. They are also used to assess risks associated with health determinants and possible beneficial effects of protective factors. Data inform the formulation, implementation and evaluation of policies, programs and actions. High-quality data is an essential requirement for measuring public health outcomes and evaluating the impact of health interventions.^6-8^

Information system performance can be measured by the quality of the data produced, but it must also consider the continued use of data to improve the health system itself and the impacts on the population’s health. Improving an information system in terms of availability, quality and use of data requires a broad set of initiatives involving technical resources, training and motivation of human resources, a favorable organizational environment and, in particular, continued use of information providing feedback to the system permanently.^6,8^

The multiplicity of information systems, as well as data collection without critical analysis or transformation into information that can be used in daily management or long-term planning, is often associated with quality problems in the collection and recording process, even in computerized systems.^6,8,9^

Vaccination coverage derived from administrative data is often assessed by comparison with coverage estimates obtained from population surveys. This approach often reveals large differences and may indicate underlying problems with data quality in one or both data sources, remembering that the accuracy of coverage based on administrative data still depends on correct estimates of the target population, introducing an additional source of error.^1,7,8^

This study chose to verify the quality of data recording for each of the vaccine doses that make up the vaccination calendar up to 24 months old, instead of simply comparing coverage between the two sources. The first aspect to draw attention was the finding that around 11% of the subsample of children selected for the study did not have any record on the computerized system (SI-PNI). As highlighted by Bloland and MacNeil,^7^ the primary data collection point, that is, the vaccination room, is generally not analyzed in system quality assessment studies, even though it potentially represents a substantial source of errors that, once recorded, are not subject to correction in higher levels of the system.^9^

The most plausible hypotheses to explain this initial loss of records would be the failure to record vaccinations carried out in private services, failure to record data on the system immediately after updating the records, given problems with the online system or staff overburdening, or even failure to correctly send municipal data to the federal level. The first hypothesis finds some support in the data presented in Table 2, showing statistically significant differences in the agreement between vaccination card data and data held on the computerized system for practically all doses analyzed, in the comparison between vaccines administered exclusively in public services and use of private services for at least one of the doses.

Bloland and MacNeil^7^ describe two indicators to characterize data quality: veracity and agreement. According to them, measuring veracity is extremely difficult in the case of vaccines, so that we are left with the option of agreement between data from different recording sources. Given that none of the sources can truly be considered “gold standard”, high agreement may suggest a greater degree of veracity.

When we consider agreement between records of dates of dose administration in the two sources ‒ vaccination card and computerized system ‒, we find two situations with different meanings: around 32% of non-recording on the computerized system of data contained on vaccination cards and around 8% of disagreement between the remaining data. In other words, when the data are in fact recorded, there tends to be a small discrepancy between them, but a significant part of the data is simply not recorded in one of the sources. Fuller found greater agreement between the computerized system and medical record data in four American states.^10^

The main reasons for the discrepancies found were errors in notation with incorrect year, month or day of dose administration, found by comparison with the children’s date of birth or by comparison with previous doses, for data held both on the vaccination cards and on the system, legibility of manual records on vaccination cards, blurs, and other aspects that make correct reading difficult.

Usability characteristics take into account the relevance of data collected, efficiency, completeness, timeliness, integrity and consistency, in addition to technical characteristics of the system itself.^7,8,11^ Regarding relevance and efficiency, the computerized system reproduces the data from vaccination cards containing only the relevant and indispensable data for coverage estimates. However, completeness is greatly compromised, with losses of children who have records, are vaccinated but were not included on the system, in addition to doses administered that were also not properly recorded, similar to the results of other studies on the SI-PNI.^8,9^ Timeliness could be compromised if the losses mentioned above were, at some point, corrected by late input to the system. Data integrity appears to be ensured for the most part, given the small percentage of disagreement for the majority of doses in the set of cities analyzed. Finally, record consistency showed a degree of variation between doses for the same municipalities and between municipalities, being more pronounced in some of them, as shown in Figure 1.

In order to improve the quality of estimates, initial concerns should focus on the point where data are initially collected. Those responsible for collecting primary data are generally overburdened, poorly motivated, seeing data recording as an undesirable task, with a lot of time spent to the detriment of other tasks. As there is little feedback on the data collected, its importance, employability and results, little time available for local analyses and a lack of understanding of the importance of recording data, problems tend to persist without solution. Without permanent use of the information generated there will be no incentive to improve data collection.^8,9^

According to data from the “National Survey on vaccination coverage, its multiple determinants and immunization actions in Brazilian municipal territories” (“*Pesquisa Nacional sobre cobertura vacinal, seus múltiplos determinantes e as ações de imunização nos territórios municipais brasileiros*”), conducted by the Núcleo de Estudos em Saúde Coletiva of the Universidade Federal de Minas Gerais,^12^ those in charge of municipal immunization programs highlighted a series of difficulties related to vaccination data recording carried out by primary care centers in their jurisdictions. 

Among the difficulties, some are particularly relevant for the data described in this study and can be grouped into difficulties in material infrastructure, scarcity and qualification of human resources and modes of information system operation. Regarding infrastructure, the following were mentioned: lack of internet access, unstable connections and lack of exclusive computers for data input or insufficient computers to guarantee timely recording of services provided. Regarding human resources, the following were highlighted: lack or insufficiency of qualified personnel to manage the system and lack of training of professionals working in vaccination rooms. Regarding how the system works: a significant proportion reported using their own local system which was incompatible with the national system, making it difficult to send data from the local to the federal level, centralized recording at the municipal level, manual recording at the point of service delivery for later input to the system and difficulty in using it to monitor the achievement of municipal vaccination targets and monitoring service users to detect problems early regarding their keeping to the vaccination schedule.^8-10^

For the SI-PNI to fulfill its objectives and provide reliable estimates of vaccination coverage, efforts will have to be made to increase data quality, ensuring that it provides a useful tool.

Comparison between vaccination card data and information system data showed that the main problem appears to be data completeness, with there being less reason to worry about the quality of the records. Underrecording of doses and children can compromise coverage estimates, altering both the numerator data and the denominator data of the calculations.

The main limitation of this study was not being able to analyze children from the birth cohorts of interest recorded on the SI-PNI who were not part of the survey sample, which would require censusing all of them, which would be unfeasible with the available resources. Taking the children included in the survey, it was only possible to follow one direction: check their records on the SI-PNI and compare them with those recorded on their vaccination cards. 

In conclusion, the results show an important problem of information underrecording on the SI-PNI that needs to be better analyzed in each state capital, seeking to identify relevant operational problems. Such underrecording negatively impacts assessments and monitoring of vaccination coverage in Brazil. It is important to separate problems with the recording system itself, which are the responsibility of the federal level of government, from the operational procedures which are the responsibility of municipal and local authorities, with the aim of improving vaccination room operations. 
