# Supplementary material for: Reliability of information recorded on the National Immunization Program Information System
Source: Epidemiol Serv Saude. 2024 Oct 21;33(spe2):e20231309. doi: 10.1590/S2237-96222024v33e20231309.especial2.en (PMC11493399; doi:10.1590/S2237-96222024v33e20231309.especial2.en)
Supplement: Translation

# Translation: Confiabilidade das informações registradas no Sistema de Informação do Programa Nacional de Imunizações

**DOI:** 10.1590/S2237-96222024v33e20231309.especial2.pt

**Authors:** José Cássio de Moraes, Ana Paula França, Ione Aquemi Guibu, Rita Barradas Barata, Grupo ICV 2020, Adriana Ilha da Silva, Alberto Novaes Ramos, Ana Paula França, Andrea de Nazaré Marvão Oliveira, Antonio Fernando Boing, Carla Magda Allan Santos Domingues, Consuelo Silva de Oliveira, Ethel Leonor Noia Maciel, Ione Aquemi Guibu, Isabelle Ribeiro Barbosa Mirabal, Jaqueline Caracas Barbosa, Jaqueline Costa Lima, José Cássio de Moraes, Karin Regina Luhm, Karlla Antonieta Amorim Caetano, Luisa Helena de Oliveira Lima, Maria Bernadete de Cerqueira Antunes, Maria da Gloria Teixeira, Maria Denise de Castro Teixeira, Maria Fernanda de Sousa Oliveira Borges, Rejane Christine de Sousa Queiroz, Ricardo Queiroz Gurgel, Rita Barradas Barata, Roberta Nogueira Calandrini de Azevedo, Sandra Maria do Valle Leone de Oliveira, Sheila Araújo Teles, Silvana Granado Nogueira da Gama, Sotero Serrate Mengue, Taynãna César Simões, Valdir Nascimento, Wildo Navegantes de Araújo

**Affiliations:** 1 Faculdade de Ciências Médicas da Santa Casa de São Paulo, Departamento de Saúde Coletiva, São Paulo, SP, Brasil; Universidade Federal do Espírito Santo, Vitória, ES, Brasil; Universidade Federal do Ceará, Departamento de Saúde Comunitária, Fortaleza, CE, Brasil; Faculdade Ciências Médicas Santa Casa de São Paulo, São Paulo, SP, Brasil; Secretaria de Estado da Saúde do Amapá, Macapá, AP, Brasil; Universidade Federal de Santa Catarina, SC, Brasil; Organização Pan-Americana da Saúde, Brasília, DF, Brasil; Instituto Evandro Chagas, Belém, PA, Brasil; Faculdade de Ciências Médicas Santa Casa de São Paulo, Departamento de Saúde Coletiva, São Paulo, SP, Brasil; Universidade Federal do Rio Grande do Norte, Natal, RN, Brasil; Universidade Federal do Ceará, Departamento de Saúde Comunitária, Fortaleza, CE, Brasil; Universidade Federal de Mato Grosso, Cuiabá, MT, Brasil; Universidade Federal do Paraná, Curitiba, PR, Brasil; Universidade Federal de Goiás, Goiânia, GO, Brasil; Universidade Federal do Piauí, Teresina, PI, Brasil; Universidade de Pernambuco, Faculdade de Ciências Médicas, Pernambuco, PE, Brasil; Instituto de Saúde Coletiva, Universidade Federal da Bahia, Salvador, BA, Brasil; Secretaria de Estado da Saúde de Alagoas, Maceió, AL, Brasil; Universidade Federal do Acre, Rio Branco, AC, Brasil; Universidade Federal do Maranhão, Departamento de Saúde Pública, São Luís, MA, Brasil; Universidade Federal de Sergipe, Aracaju, SE, Brasil; Secretaria Municipal de Saúde, Boa Vista, RR, Brasil; Fundação Oswaldo Cruz, Mato Grosso do Sul, Campo Grande, MS, Brasil; Fundação Oswaldo Cruz, Escola Nacional de Saúde Pública Sergio Arouca, Rio de Janeiro, RJ, Brasil; Universidade Federal do Rio Grande do Sul, Porto Alegre, RS, Brasil; Fundação Oswaldo Cruz, Instituto de Pesquisa René Rachou, Belo Horizonte, MG, Brasil; Secretaria de Desenvolvimento Ambiental de Rondônia, Porto Velho, RO, Brasil; Universidade de Brasília, Brasília, DF, Brasil

**Keywords:** Sistema de Informação em Saúde, Confiabilidade, Vacinas

## Abstract

**Objetivo:**

Analisar a confiabilidade dos registros no Sistema de Informação do Programa Nacional de Imunizações (SI-PNI) em uma subamostra de crianças incluídas no inquérito nacional de cobertura vacinal nas capitais brasileiras e no Distrito Federal, em 2020.

**Método:**

Estudo de concordância entre registros nas cadernetas (doses e datas) e no SI-PNI para 4.050 crianças com esquema completo aos 24 meses.

**Resultados:**

Foram localizados registros de 3.587 crianças no SI-PNI, havendo 11% (IC_95_%10,0;12,0) de perdas. A concordância total entre doses e datas nas duas fontes foi de 86% (IC_95_% 86,0;87,0), porém para cada dose e vacina a variação foi maior, com 32% de dados só em uma fonte.

**Conclusão:**

Parte das informações não vem sendo adequadamente registrada, mas para os dados existentes nas duas fontes a discordância pode ser considerada pequena. O sub-registro de doses e crianças pode comprometer as estimativas de cobertura vacinal, alterando os dados do numerador e do denominador.

## Contribuições do estudo

**Principais resultados:**

Na subamostra de 4.050 crianças, entre aquelas com esquema completo aos 24 meses estudadas no inquérito nacional, 11% não estavam registradas no SI-PNI, 32% tinham doses não registradas (doses ou datas) e 8% discordantes entre caderneta e SI-PNI.

**Implicações para os serviços:**

Reconhecer as dificuldades do SI-PNI e as discrepâncias entre as fontes é fundamental para a adoção de iniciativas de melhoria da qualidade dos dados, evitandose as estimativas imprecisas da cobertura vacinal na infância.

**Perspectivas:**

A partir deste estudo, esperase contribuir para aprimorar a qualidade dos registros e a usabilidade dos dados para o acompanhamento da cobertura vacinal, desde o nível local até o nível nacional do programa de imunizações.

## INTRODUÇÃO

O Programa Nacional de Imunizações (PNI) é o elemento central no controle das doenças imunopreveníveis, aquele conjunto de enfermidades para as quais as vacinas foram desenvolvidas como instrumentos de prevenção primária, ou seja, capazes de impedir a infecção ou o desenvolvimento da doença nos imunizados. O arranjo tecnológico adotado no controle desse grupo de doenças transmissíveis combina uma série de elementos, como a vacinação de rotina, os dias nacionais de vacinação, as campanhas eventuais e a vigilância epidemiológica.^1^

A vacinação de rotina consiste no estabelecimento de um calendário nacional de vacinação, contendo todas as vacinas que devem ser oferecidas aos diferentes grupos etários da população, a indicação do número de doses para cada imunizante, bem como as idades corretas de aplicação (adequação epidemiológica) e os intervalos corretos entre as doses (adequação imunológica). A intenção é obter coberturas populacionais suficientes para que a imunidade de grupo ou de rebanho possa funcionar efetivamente como um mecanismo de proteção também para aqueles que não foram vacinados, mantendo a circulação dos agentes etiológicos sob controle.^1^

Os dias nacionais de vacinação, organizados sob a forma de uma ação massiva realizada duas vezes ao ano para ampliar e facilitar o acesso àquelas famílias que, por diferentes razões, têm dificuldades no uso regular dos serviços de saúde, representaram, durante muitos anos, uma estratégia de discriminação positiva, visando à oferta equitativa de oportunidades para vacinar todos os grupos populacionais do país, de forma complementar às atividades de rotina.^2^

As campanhas de vacinação eventuais ou a chamada vacinação de bloqueio ocorrem diante da identificação de surtos ou da ocorrência de casos de doenças evitáveis por vacinação, em conglomerados espaço-temporais delimitados, funcionando como um mecanismo supletivo na proteção de grupos ou segmentos populacionais particulares. Geralmente essas ações estão articuladas ao sistema de vigilância epidemiológica, que, ao detectar casos suspeitos ou confirmados dessas doenças, aciona diversas ações de contenção para interromper a transmissão das doenças.

O acompanhamento da situação vacinal do grupo das crianças menores de 24 meses é particularmente importante, pela relevância que as doenças imunopreveníveis têm na morbidade e mortalidade na infância, tanto nos menores de 1 ano (mortalidade infantil) quanto nos menores de 5 anos. Assim, a informação sobre cobertura vacinal na infância é essencial para as análises de situação de saúde e o direcionamento das políticas de saúde da criança.

O SI-PNI é formado por um conjunto de sistemas, de modo a atender a todos os aspectos envolvidos na realização do programa nacional de imunizações: avaliação do programa de imunizações, estoque e distribuição de imunobiológicos, eventos adversos pós-vacinação, programa de avaliação do instrumento de supervisão, programa de avaliação do instrumento de supervisão em sala de vacinação, apuração dos imunobiológicos utilizados e sistema de informações dos Centros de Referência em Imunobiológicos Especiais.^3^ Apenas o sistema de avaliação do programa de imunizações será objeto deste estudo.

Antes da criação do SI-PNI, a cobertura vacinal era expressa pelo número de doses aplicadas para uma determinada vacina sobre o número registrado ou estimado de menores de 1 ano ou menores de 24 meses existentes na área de abrangência do programa. Essa forma de estimar a cobertura habitualmente gerava valores superestimados para cada imunizante, não permitindo avaliar a cobertura pelo esquema completo, uma vez que as informações tinham vários graus de imprecisão, além de não corresponderem à experiência de cada uma das crianças no grupo etário de interesse.^1^

O SI-PNI foi desenhado para permitir o acompanhamento longitudinal de cada criança incluída no programa, além de permitir que vacinas aplicadas em diferentes serviços de saúde sejam registradas na mesma caderneta-espelho, valendo-se para tanto de um registro *on-line*. Cada uma das doses de vacina deve ser registrada na caderneta de vacinação da criança e no sistema de informação, garantindo-se um registro fidedigno e redundante das doses aplicadas, fundamental para aumentar a segurança da manutenção do histórico vacinal do indivíduo. Além de fornecer uma ferramenta de análise da cobertura vacinal em diferentes níveis de análise (serviço de saúde, distrito, município, estado e país), o sistema permite a recuperação do histórico de vacinação de cada criança mesmo que o documento físico, a caderneta de vacinação seja perdido ou danificado.^4^

A introdução do novo sistema de informação, iniciada em 2010, progressivamente alcançou cobertura nos cinco anos seguintes, coincidindo com um período de redução das coberturas vacinais infantis no país, gerando-se um conjunto de dúvidas sobre os motivos para tal redução, incluindo o possível papel do novo sistema de informações. Tendo em vista a dimensão geográfica do país e as dificuldades conhecidas, relativas às redes de internet em diferentes regiões e a lentidão que um sistema nacional pode apresentar no momento de registrar as informações, uma das questões levantadas era o possível atraso no registro e envio das informações desde o nível local, acarretando aparente redução de coberturas.

A ocorrência das epidemias de sarampo, entretanto, pareciam indicar uma redução real nas coberturas, independentemente de problemas no sistema de registro. Desse modo, era necessário avaliar até que ponto o registro no SI-PNI estava sendo adequadamente realizado.

O objetivo deste estudo foi analisar a confiabilidade dos registros realizados no sistema de avaliação do programa de imunizações, parte do SI-PNI, para uma subamostra de crianças incluídas no inquérito nacional de cobertura vacinal, realizado nas capitais brasileiras e no Distrito Federal, em 2020, com cobertura completa aos 24 meses de idade. As perguntas de investigação foram: 

*Quantas destas crianças estavam registradas no SI-PNI? Para as registradas, qual era a concordância entre os dados da caderneta e os dados do SI-PNI?*

## MÉTODOS

A realização do inquérito nacional de cobertura vacinal nas capitais brasileiras e no Distrito Federal ofereceu a oportunidade de analisar uma subamostra das crianças nascidas em 2017 ou em 2018, residentes nas áreas urbanas dessas cidades, comparando-se os dados disponíveis nas cadernetas de vacinação com aqueles do SI-PNI. A metodologia do inquérito está descrita, em detalhes, em outra publicação.^5^

Para este estudo de confiabilidade, foram consideradas como população-alvo as crianças participantes do inquérito nacional que apresentaram cobertura completa para as vacinas previstas até os 24 meses de idade, conforme documentação, doses e datas de aplicação, na caderneta de vacinação. Portanto, o registro na caderneta foi considerado o padrão ouro. As cadernetas foram fotografadas durante a entrevista domiciliar e posteriormente os dados foram transcritos para os questionários de cada criança, sob a supervisão de um profissional de enfermagem com experiência no PNI.

Das 31.001 crianças incluídas no inquérito nacional, 18.808 (58%) apresentavam a cobertura vacinal completa. Desse total, sortearam-se 150 crianças residentes em cada uma das capitais e no Distrito Federal, obtendo-se uma subamostra de 4.050 crianças. Essa estratégia permitiu realizar comparações na qualidade do preenchimento dos dados no SI-PNI entre as capitais.

O tamanho da amostra foi definido de forma arbitrária, tendo em vista o trabalho implicado na localização das crianças no SI-PNI e na transcrição dos dados para posterior análise.

Para cada uma das 4.050 crianças da subamostra, foram buscados os registros correspondentes no SI-PNI, a partir do nome da mãe e da data de nascimento da criança. Foram registradas no banco de dados tanto as doses e datas presentes na caderneta de vacinação quanto os registros correspondentes no SI-PNI. 

Os dados foram comparados quanto ao registro da dose e as datas constantes nas duas fontes de informação. Para cada dose, os resultados foram classificados em três grupos: concordância das datas registradas em ambos os instrumentos; discordância entre as datas registradas em um dos instrumentos; e registro existente apenas na caderneta de vacinação, não constando da ficha espelho. Foram calculados os percentuais e intervalos de confiança de 95% (IC 95%).

Foram comparados os percentuais de concordância entre doses e datas nas duas fontes de informação, separadamente, para crianças que utilizaram serviços públicos de vacinação e para aquelas que receberam pelo menos uma dose em serviços privados. A associação foi testada pelo qui-quadrado.

A distribuição dos percentuais de concordância entre os dados nas duas fontes, para cada uma das doses de vacinas aplicadas a cada criança, em cada cidade, foi analisada por meio de gráficos *boxplot*. Os gráficos *boxplot* apresentam um resumo da distribuição representando o valor mediano e o intervalo interquartílico nas caixas (*box*) e os valores discrepantes, para mais ou para menos, em sinais gráficos apresentados fora das caixas. Assim, foi possível comparar os valores observados em cada capital quanto à concordância entre as duas fontes de informação.

Para cada uma das 150 crianças em cada cidade, foi analisada a concordância combinada de doses que compõem o esquema preconizado para os primeiros 24 meses de vida. Foram consideradas as três doses da vacina pentavalente (considerando-se todas as apresentações disponíveis), as três doses da vacina contra a poliomielite inativada, as duas doses e o reforço da vacina antipneumocócica, as duas doses e o reforço da vacina antimeningocócica C, as duas doses da vacina contra o rotavírus, a primeira dose da vacina tríplice viral, as vacinas contra a hepatite A e contra a varicela. Não foram incluídas as vacinas Bacilo de Calmette-Guérin (BCG) e hepatite B, ao nascer, porque os registros no SI-PNI foram ausentes para a maioria das crianças, e febre amarela, porque não estava implementada em todas as cidades em 2017 e 2018. A segunda dose da tríplice viral também foi excluída, por apresentar importante ausência de informação no SI-PNI.

Todas as análises e a construção dos gráficos foram feitas no programa Stata versão 17.0.

O estudo foi aprovado pelos Comitês de Ética em Pesquisa com Seres Humanos do Instituto de Saúde Coletiva da Universidade Federal da Bahia, sob parecer n° 3.366.818, em 4 de junho de 2019, com Certificado de Apresentação de Apreciação Ética (CAAE) 4306919.5.0000.5030; e da Irmandade da Santa Casa de São Paulo, sob parecer n° 4.380.019, em 4 de novembro de 2020, com CAAE 39412020.0.0000.5479. 

## RESULTADOS

Das 4.050 crianças sorteadas, 3.587 (89%) tiveram a ficha espelho localizada no SI-PNI. Portanto, 11% (IC_95_%10,0;12,0) das crianças não tinham seus dados registrados no sistema. As perdas ocorreram em todas as cidades, estando acima de 20% em São Luís, Rio de Janeiro e Campo Grande (Tabela 4).

A Tabela 1 apresenta, para cada dose de vacina, a proporção de doses com registros concordantes, discordantes ou ausentes no SI-PNI para o total de 3.587 crianças com informações em ambas as fontes de dados. De modo geral, cerca de 32% das informações de aplicação das doses estavam ausentes no SI-PNI, 58% a 60% das informações eram concordantes em ambas as fontes e 8% eram discordantes. Ainda nesta tabela, chamam a atenção os percentuais bem mais altos de ausência de registros eletrônicos para as doses de BCG intradérmica, hepatite B e tríplice viral (segunda dose). 

**Tabela 1:** Concordância (%) entre as informações da caderneta de vacinação e o registro no SI-PNI, segundo dose de vacina do calendário vacinal até os 24 meses de idade, Inquérito Nacional de Cobertura Vacinal, 2020

Vacina	Concordante N (%)	Discordante N (%)	Ausente N (%)
Bcg intradérmica	1.239 (34,5)	123 (3,4)	2.223 (62,1)
Hepatite B	999 (27,9)	135 (3,8)	2.449 (68,3)
Pentavalente 1^a^ dose	2.211 (61,6)	218 (6,1)	1.155 (32,3)
Pentavalente 2^a^ dose	2.177 (60,7)	254 (7,1)	1.147 (32,2)
Pentavalente 3^a^ dose	2.176 (60,7)	277 (7,7)	1.077 (31,6)
Poliomielite inativada 1ª dose	2.276 (63,4)	207 (5,8)	1.104 (30,8)
Poliomielite inativada 2ª dose	2.220 (61,9)	249 (6,9)	1.115 (31,2)
Poliomielite inativada 3ª dose	2.177 (60,7)	262 (7,3)	1.099 (32,0)
Pneumocócica 1ª dose	2.166 (60,4)	285 (7,9)	1.136 (31,7)
Pneumocócica 2ª dose	2.138 (59,6)	283 (7,9)	1.166 (32,5)
Pneumocócico reforço	2.230 (62,1)	296 (8,3)	1.056 (29,6)
Rotavírus 1^a^ dose	2.232 (62,3)	252 (7,0)	1.075 (30,7)
Rotavírus 2^a^ dose	2.148 (59,9)	242 (6,7)	1.149 (33,4)
Meningocócica 1ª dose	2.180 (60,8)	327 (9,1)	1.080 (30,1)
Meningocócica 2ª dose	2.107 (58,8)	320 (8,9)	1.157 (32,3)
Meningocócico reforço	2.208 (61,5)	282 (7,9)	995 (30,6)
Hepatite A	2.401 (66,9)	250 (7,0)	934 (26,1)
Tríplice viral 1^a^ dose	2.344 (65,4)	536 (14,9)	706 (19,7)
Tríplice viral 2^a^ dose	951 (26,5)	134 (3,7)	619 (69,8)
Varicela	2.344 (65,4)	262 (7,3)	971 (27,3)
Poliomielite oral 1^o^ reforço	2.255 (62,8)	401 (11,2)	878 (26,0)
Tríplice bacteriana 1^o^ reforço	2.088 (58,2)	297 (8,3)	953 (33,5)

A Tabela 2 apresenta a comparação dos registros concordantes para crianças que receberam todas as doses nos serviços públicos e para as que receberam pelo menos uma dose nos serviços privados. De modo geral, houve maior concordância quando as vacinas foram aplicadas nos serviços públicos, exceção feita para a BCG, primeira dose da vacina para o rotavírus, e segunda dose da vacina tríplice viral. Para a vacina contra a hepatite B, embora a concordância estimada tenha sido maior entre as crianças que usaram serviços privados, a diferença não foi estatisticamente significante.

**Tabela 2:** Concordância (%) entre as informações da caderneta de vacinação e o registro no SI-PNI, segundo dose de vacina do calendário vacinal até os 24 meses de idade, para crianças vacinadas exclusivamente ou não nos serviços públicos, Inquérito Nacional de Cobertura Vacinal, 2020

Vacinas	Recebidas exclusivamente nos serviços públicos		p-valor
	Não	Sim	
Bcg intradérmica	279 (39,9)	938 (33,2)	0,026
Hepatite B	219 (31,3)	766 (27,1)	0,202
Pentavalente 1^a^ dose	392 (56,0)	1.786 (63,2)	0,001
Pentavalente 2^a^ dose	376 (53,7)	1.771 (62,7)	< 0,001
Pentavalente 3^a^ dose	351 (50,1)	1.790 (63,4)	< 0,001
Poliomielite inativada 1ª dose	419 (59,9)	1.822 (64,5)	0,005
Poliomielite inativada 2ª dose	389 (55,6)	1.797 (63,6)	< 0,001
Poliomielite inativada 3ª dose	362 (51,7)	1.784 (63,2)	< 0,001
Pneumocócica 1ª dose	394 (56,3)	1.741 (61,7)	0,030
Pneumocócica 2ª dose	393 (56,1)	1.711 (60,6)	0,011
Pneumocócico reforço	365 (37,1)	1.828 (64,7)	< 0,001
Rotavírus 1ª dose	418 (58,4)	1.782 (63,1)	0,007
Rotavírus 2ª dose	409 (58,4)	1.704 (60,3)	0,005
Meningocócica 1ª dose	394 (56,3)	1.751 (62,0)	0,042
Meningocócica 2ª dose	370 (52,9)	1.706 (60,4)	< 0,001
Meningocócica reforço	323 (46,1)	1.846 (65,4)	< 0,001
Hepatite A	437 (62,4)	1.925 (68,2)	0,045
Tríplice viral 1ª dose	428 (61,1)	1.882 (66,6)	0,017
Tríplice viral 2ª dose	207 (29,6)	727 (25,7)	< 0,001
Varicela	421 (60,1)	1.887 (66,8)	0,019
Poliomielite oral 1^o^ reforço	398 (56,9)	1.819 (64,4)	< 0,001
Tríplice bacteriana 1^o^ reforço	331 (47,3)	1.726 (61,1)	< 0,001

A Figura 1 apresenta a distribuição das proporções de concordância para as doses analisadas em cada uma das capitais e no Distrito Federal. Os municípios foram agrupados por região, para se tornar mais fácil a visualização. Pode-se observar a grande variação entre os municípios da região Nordeste, com maior concordância em geral observada para Teresina, Fortaleza, Natal, Aracaju e Salvador, e a menor, para São Luís e João Pessoa.

**Figura 1:** Distribuição da concordância (%) para cada vacina e dose segundo as capitais e o Distrito Federal, em cada região brasileira, Inquérito Nacional de Cobertura Vacinal, 2020

Na região Norte, Palmas e Macapá apresentavam maior concordância, e os menores valores foram observados em Boa Vista, havendo grande variabilidade nessa cidade. Na região Sudeste, a situação foi mais homogênea, com exceção do Rio de Janeiro, com menor concordância em geral. Na região Sul, Florianópolis apresentou a menor concordância e a maior dispersão. No Centro-Oeste, o pior desempenho foi observado em Campo Grande, ficando Cuiabá em posição intermediária e Goiânia e Brasília com concordância maior.

A Tabela 3 mostra as proporções extremamente baixas de concordância para o conjunto de vacinas selecionadas em cada uma das capitais e no Distrito Federal, quando observados longitudinalmente os dados de cada criança. Esses dados sugerem que os cálculos de cobertura pelo esquema completo dariam resultados distintos, dependendo da fonte de informações utilizada.

**Tabela 3:** Registros ausentes (%) do SI-PNI e concordância (%) entre as informações da caderneta de vacinação e o registro no SI-PNI para o conjunto das vacinas^a^ do calendário vacinal até os 24 meses de idade, por capitais e Distrito Federal, Inquérito Nacional de Cobertura Vacinal, 2020

Cidade	Registros ausentes (IC_95_ %)	Concordância (IC_95_ **%**)
**Região Norte**		
Macapá	11,3 (7,0;17,2)	9,8 (5,0;14,5)
Belém	9,3 (5,4;14,8)	12,5 (7,2;17,8)
Rio Branco	4,7 (2,0;9,9)	13,3 (7,8;18,7)
Boa Vista	6,7 (3,4;11,6)	14,3 (8,7;19,9)
Manaus	6,0 (3,0;10,7)	16,3 (10,4;22,2)
Palmas	6,0 (3,0;10,7)	17,0 (11,0;23,0)
Porto Velho	3,3 (1,2;7,2)	17,9 (11,8;24,1)
**Região Nordeste**		
Teresina	12,0 (7,5;18,0)	9,8 (5,1;14,6)
Aracaju	10,0 (6,4;16,6)	10,4 (5,5;15,2)
João Pessoa	16,7 (11,3;23,3)	11,2 (6,1;16,2)
Fortaleza	6,7 (3,4;11,7)	12,8 (7,5;18,2)
Recife	10,7 (6,4;16,5)	13,4 (8,0;18,9)
Maceió	10,7 (6,4;16,5)	14,2 (8,6;19,8)
Salvador	10,7 (6,4;16,5)	15,7 (9,8;21,5)
Natal	17,3 (11,9;24,0)	16,1 (10,2;22,0)
São Luís	26,0 (19,5;33,5)	19,8 (13,4;26,2)
**Região Sudeste**		
Belo Horizonte	8,0 (4,4;13,2)	10,1 (5,3;15,0)
Rio de Janeiro	22,7 (16,5;30,0)	10,3 (5,5;15,2)
São Paulo	17,3 (11,9;24,1)	16,1 (10,2;22,0)
Vitória	16,7 (11,3;23,4)	19,2 (12,9;25,5)
**Região Sul**		
Porto Alegre	10,7 (6,4;16,7)	11,3 (6,2;16,3)
Curitiba	9,3 (5,4;14,9)	16,2 (10,3;22,1)
Florianópolis	10,0 (5,9;15,7)	16,3 (10,4;22,2)
**Região Centro-Oeste**		
Cuiabá	7,3 (3,9;12,4)	7,2 (3,1;11,4)
Goiânia	11,3 (7,0;17,3)	14,3 (8,7;19,9)
Campo Grande	24,0 (17,7;31,3)	19,3 (13,0;25,6)
**Distrito Federal**	3,3 (1,2;7,3)	15,2 (9,4;20,9)

a) Vacinas: pentavalente, pólio inativada, pneumocócica, rotavírus, meningocócica C, tríplice viral, hepatite A e varicela.

## DISCUSSÃO

Em saúde pública, dados são usados para monitorar tendências e comportamentos das distribuições populacionais de eventos de interesse, tais como casos, óbitos, exposições, características sociodemográficas, entre outros. Eles também são usados para avaliar os riscos associados aos determinantes de saúde e possíveis efeitos benéficos de fatores protetores. Os dados informam a formulação, implementação e avaliação de políticas, programas e ações. Dados de alta qualidade são requisitos indispensáveis para mensurar resultados em saúde pública e avaliar o impacto das intervenções em saúde.^6-8^

A *performance* do sistema de informação pode ser medida pela qualidade dos dados produzidos, mas também deve considerar o uso continuado dos dados para melhorar o próprio sistema de saúde e os impactos sobre a saúde populacional. Melhorar um sistema de informação em termos de disponibilidade, qualidade e uso dos dados requer um conjunto amplo de iniciativas envolvendo recursos técnicos, treinamento e motivação dos recursos humanos, ambiente organizacional favorável, e principalmente uso continuado das informações, fornecendo retroalimentação para o sistema permanentemente.^6,8^

 A multiplicidade de sistemas de informação, bem como a coleta de dados sem análise crítica ou transformação em informações que possam ser usadas na gestão cotidiana ou em planejamento de longo prazo, muitas vezes se associa a problemas de qualidade no processo de coleta e registro, mesmo em sistemas informatizados.^6,8,9^ As coberturas vacinais derivadas de dados administrativos são frequentemente avaliadas por comparação com estimativas de cobertura obtidas em inquéritos populacionais. Essa abordagem geralmente revela grandes diferenças, podendo indicar problemas subjacentes com a qualidade dos dados em uma ou em ambas as fontes de dados, ressaltando-se que a exatidão da cobertura baseada em dados administrativos ainda depende das estimativas corretas da população-alvo, introduzindo uma fonte adicional de erro.^1,7,8^

Este estudo optou por verificar a qualidade do registro dos dados de cada uma das doses de vacina que compõem o calendário vacinal até os 24 meses de idade, em vez de simplesmente comparar as coberturas entre as duas fontes. O primeiro aspecto a chamar a atenção foi a constatação de que cerca de 11% da subamostra de crianças selecionadas para o estudo não tinham nenhum registro no sistema informatizado (SI-PNI). Como destacado em artigo sobre imunização,^7^ o ponto de coleta primário dos dados, ou seja, a sala de vacina, geralmente não é analisado nos estudos de avaliação de qualidade dos sistemas, ainda que potencialmente represente uma fonte substancial de erros que, uma vez registrados, não são passíveis de correção em níveis mais altos do sistema.^9^

As hipóteses mais plausíveis para explicar essa perda inicial de registros seriam o não registro da vacinação conduzida nos serviços privados, o não registro de dados no sistema imediatamente após a atualização das cadernetas, tendo em vista problemas com o sistema *on-line* ou excesso de tarefas dos servidores, ou ainda o não envio correto dos dados municipais para o nível federal. A primeira hipótese encontra algum respaldo nos dados apresentados na Tabela 2, que mostra diferenças estatisticamente significantes na concordância entre os dados da caderneta e do sistema informatizado para praticamente todas as doses analisadas, na comparação entre vacinas aplicadas exclusivamente nos serviços públicos e a utilização de serviços privados para aplicação de pelo menos uma das doses.

O estudo citado anteriormente^7^ descreve dois indicadores para caracterizar a qualidade dos dados: a veracidade e a concordância. Segundo o estudo, a mensuração da veracidade é extremamente difícil no caso das vacinas, restando então a concordância entre dados de diferentes fontes de registro, tendo em vista que nenhuma das fontes pode ser realmente considerada padrão ouro, a concordância alta pode sugerir maior grau de veracidade.

Quando consideramos a concordância entre registros de datas da aplicação das doses nas duas fontes ‒ caderneta e sistema informatizado ‒, encontramos duas situações com significados diferentes: cerca de 32% de não registro no sistema informatizado de dados constantes na caderneta e cerca de 8% de discordância entre os dados restantes. Ou seja, quando os dados estão registrados, tende a haver pequena discrepância entre eles, porém parte significativa dos dados simplesmente não estão registrados em uma das fontes. Fuller encontrou maior concordância entre o sistema informatizado e os dados de prontuários em quatro estados norte-americanos.^10^

Os principais motivos para as discordâncias observadas foram erros de anotação, com troca do ano, do mês ou do dia de aplicação, observados por comparação com a data de nascimento das crianças ou ainda por comparação com as doses anteriores, presentes tanto nas cadernetas quanto no sistema, legibilidade dos registros manuais nas cadernetas, borrões, e outros aspectos que dificultam a leitura correta.

As características de usabilidade levam em conta a relevância dos dados coletados, a eficiência, a completude, a oportunidade, a integridade e a consistência, além de características técnicas do próprio sistema.^7,8,11^ Quanto à relevância e eficiência, o sistema informatizado reproduz os dados da caderneta, contendo apenas os dados relevantes e indispensáveis para as estimativas das coberturas. Mas, a completude está bastante comprometida, com perdas de crianças que possuem cadernetas, estão vacinadas e não foram incluídas no sistema, além de doses aplicadas que também não foram registradas devidamente, à semelhança de resultados de outros estudos sobre o SI-PNI.^8,9^ Já a oportunidade poderia estar comprometida, caso as perdas anteriormente referidas fossem, em algum momento, compensadas por registros tardios. A integridade dos dados parece estar em grande parte preservada, dado o pequeno percentual de discordância para a maioria das doses no conjunto das cidades analisadas. Finalmente, a consistência de registros apresentou alguma variação entre doses para os mesmos municípios e entre municípios ,sendo mais pronunciadas em alguns deles, como mostrado na Figura 1.

Para melhorar a qualidade das estimativas, as preocupações iniciais devem focar o ponto em que os dados são inicialmente coletados. Os responsáveis pela coleta de dados primários estão geralmente sobrecarregados, pouco motivados, vendo o registro de dados como uma tarefa indesejável, com grande gasto de tempo desviado de outras tarefas. Como há pouco *feedback* sobre os dados coletados, sua importância, empregabilidade e resultados, pouco tempo disponível para as análises locais e falta de compreensão da importância da tarefa de registro, os problemas tendem a persistir sem solução. Sem o uso permanente das informações geradas, não haverá incentivo para a melhoria da coleta.^8,9^

Segundo dados da “Pesquisa Nacional sobre cobertura vacinal, seus múltiplos determinantes e as ações de imunização nos territórios municipais brasileiros”, realizada pelo Núcleo de Estudos em Saúde Coletiva da Universidade Federal de Minas Gerais,^12^ os responsáveis pelos programas municipais de imunização destacaram uma série de dificuldades relacionadas ao registro dos dados de vacinação realizados pelas unidades básicas de saúde em suas jurisdições. 

Entre as dificuldades, algumas têm especial relevância para os dados descritos neste estudo, e podem ser agrupadas em dificuldades de infraestrutura material, escassez e qualificação de recursos humanos e modos de operação do sistema de informações. Com relação à infraestrutura, foram mencionadas: falta de acesso à internet; instabilidade das conexões e falta de computadores exclusivos para a atividade ou insuficiência de computadores para garantir o registro oportuno dos atendimentos. Quanto aos recursos humanos, foram destacados: falta ou insuficiência de pessoal qualificado para o manejo do sistema; e falta de capacitação dos profissionais que atuam nas salas de vacina. Quanto ao modo de funcionamento do sistema: proporção expressiva relatou utilizar sistema próprio incompatível com o sistema nacional dificultando o envio dos dados do nível local para o federal; o registro centralizado no âmbito municipal; a realização de registro manual no ponto de entrega da ação para posterior digitação no sistema; e dificuldades de uso para monitorar o alcance das metas de vacinação municipais, bem como o próprio acompanhamento dos usuários, para detectar precocemente problemas no cumprimento do calendário.^8-10^

Para que o SI-PNI possa cumprir seus objetivos e fornecer estimativas confiáveis de cobertura vacinal, esforços terão que ser feitos visando aumentar a qualidade dos dados, garantindo uma ferramenta útil.

A comparação entre os dados da caderneta e aqueles registrados no sistema de informação mostrou que o principal problema parece ser o de completude dos dados, havendo menos motivo de preocupação com a qualidade dos registros. O sub-registro de doses e de crianças pode comprometer as estimativas de cobertura, alterando tanto os dados do numerador quanto os dados do denominador dos cálculos.

A principal limitação do estudo foi não poder analisar as crianças das coortes de nascimento de interesse registradas no SI-PNI que não fizeram parte da amostra do inquérito, o que exigiria recensear todas elas, e isso seria inviável com os recursos disponíveis. Partindo das crianças incluídas no inquérito, só foi possível seguir uma direção: verificar seus registros no SI-PNI e compará-los com aqueles das cadernetas de vacinação. 

Concluindo, os resultados mostram um importante problema de sub-registro de informações no SI-PNI que precisa ser melhor analisado em cada capital, buscando-se identificar os problemas operacionais relevantes. Esse sub-registro impacta negativamente as avaliações e o acompanhamento das coberturas vacinais no país. É importante separar problemas no sistema de registro em si, de responsabilidade da esfera federal, dos procedimentos operacionais de responsabilidade das autoridades municipais e locais, visando aprimorar o funcionamento das salas de vacina.
